# Phytochemical, antioxidant and protective effect of cactus cladodes extract against lithium-induced liver injury in rats

**DOI:** 10.1080/13880209.2016.1255976

**Published:** 2016-12-12

**Authors:** Anouar Ben Saad, Brahmi Dalel, Ilhem Rjeibi, Amani Smida, Sana Ncib, Nacim Zouari, Lazhar Zourgui

**Affiliations:** a Research Unit of Macromolecular Biochemistry and Genetics, Faculty of Sciences of Gafsa, University of Gafsa, Gafsa, Tunisia;; b Research Unit of Active Biomolecules Valorisation, High Institute of Applied Biology of Medenine, University of Gabes, Medenine, Tunisia;; c Laboratory of Research on Biologically Compatible Compounds, Faculty of Dental Medicine, University of Monastir, Gafsa, Tunisia;; d Common Services Unit, Faculty of Sciences Gafsa, University of Gafsa, Gafsa, Tunisia;; e High Institute of Applied Biology of Medenine, University of Gabes, Medenine, Tunisia

**Keywords:** Alkali metal, oxidative stress, histopathology, liver damage

## Abstract

**Context:** *Opuntia ficus-indica* (L.) Mill. (Castaceae) (cactus) is used in Tunisian medicine for the treatment of various diseases.

**Objective:** This study determines phytochemical composition of cactus cladode extract (CCE). It also investigates antioxidant activity and hepatoprotective potential of CCE against lithium carbonate (Li_2_CO_3_)-induced liver injury in rats.

**Materials and methods:** Twenty-four Wistar male rats were divided into four groups of six each: a control group given distilled water (0.5 mL/100 g b.w.; i.p.), a group injected with Li_2_CO_3_ (25 mg/kg b.w.; i.p.; corresponding to 30% of the LD_50_) twice daily for 30 days, a group receiving only CCE at 100 mg/kg of b.w. for 60 days and then injected with distilled water during the last 30 days of CCE treatment, and a group receiving CCE and then injected with Li_2_CO_3_ during the last 30 days of CCE treatment. The bioactive components containing the CCE were identified using chemical assays.

**Results:** Treatment with Li_2_CO_3_ caused a significant change of some haematological parameters including red blood cells (RBC), white blood cells (WBC), haemoglobin content (Hb), haematocrit (Ht) and mean corpuscular volume (VCM) compared to the control group. Moreover, significant increases in the levels of glucose, cholesterol, triglycerides and of aspartate aminotransferase (AST), alanine aminotransferase (ALT), alkaline phosphatase (ALP) and lactate dehydrogenase (LDH) activities were observed in the blood of Li_2_CO_3_-treated rats. Furthermore, exposure to Li_2_CO_3_ significantly increased the LPO level and decreased superoxide dismutase (SOD), catalase (CAT) and glutathione peroxidase (GPx) activities in the hepatic tissues.

**Conclusion:** CCE possesses a significant hepatoprotective effect.

## Introduction

Lithium (Li) is widely used in the treatment of bipolar disorder and has received a great deal of attention in the existing research literature (Sharif et al. 2011). Lithium is initially distributed in the extracellular fluid and then accumulates in some major organs, such as the liver and kidney. Prolonged treatment with Li was associated with many diseases, including diabetes insipidus (Sahu et al. 2013), thyroid, goitre development (Rogers & Whybrow 1971) and haematological dysfunctions (Oktem et al. 2005). On the other hand, it has been reported that lithium inflicts oxidative damage on liver by generating reactive oxygen species (ROS) increasing lipid peroxidation (Nciri et al. 2012), and reducing antioxidant enzyme activities (SOD, CAT and GPx) (Vijaimohan et al. 2010).

In the recent years, alternative therapeutic approaches have become very popular (Narayana & Dobriyalm 2000). Nature has been a source of medicinal treatments for thousands of years (King et al. 1998). Previous studies indicate that a great number of medicinal plants have the ability to biosynthesize phytochemicals, possessing several activities that are used to exert an efficient protective effect against oxidative stress and related disease. The cactus [*Opuntia ficus-indica* (L.) Mill. (Cactaceae)] is widely grown in Latin America, South Africa and the Mediterranean area. According to several studies, cactuses are known for their high content of fibre, mineral, vitamins, fatty acids and carotenoids (Tesoriere et al. 2005). It can be used as an antidiabetic, ulcerogenic, antiviral, anti-inflammatory and analgesic agent, and may protect against numerous chronic diseases (Wiese et al. 2004; Alimi et al. 2011; Antunes-Ricado et al. 2015).

To the best of our knowledge, there are no data concerning the *in vivo* effect of cactus *Opuntia ficus-indica* cladodes extract on hepatic damage and oxidative stress induced by lithium. Thus, the present study, carried out in rats, evaluates the protective effect of CCE against lithium-induced oxidative stress and hepatotoxicity through an analysis of serum biochemical assay, antioxidant enzyme activities in liver and histopathology analysis.

## Materials and methods

### Plant material and preparation of extract

Cactus young cladodes (2–3 weeks of age) were collected at the beginning of March 2014 in Gafsa, state of Tunisia. A voucher specimen (OFI 0214) was identified and authenticated by a taxonomist, Dr. Boulbaba Ltayef, and deposited at the herbarium (H03) in the Faculty of Sciences, University of Gafsa, Tunisia. The sample was washed with water, cut into small pieces and then pressed using a hand-press, homogenized with 10 mM Tris-HCl, pH 7.4 at 4 °C and centrifuged 30 min at 3500*g* at 4 °C. The supernatant was subsequently collected and lyophilized. Before use, the lyophilized extract was dissolved in water.

### Phytochemical study of Opuntia ficus indica

#### Determination of total phenolic and flavonoid content

The total phenolic content of CCE was determined by the Folin–Ciocalteu method (Dewanto et al. 2002). Total phenolic content was expressed as mg gallic acid equivalent (GAE)/g extract and gallic acid was used as standard. The total flavonoid content of the samples was determined as previously described (Dewanto et al. 2002) and quercetin was used as standard. The results were expressed as mg quercetin equivalent (QE)/g extract.

#### Extraction of CCE polysaccharides

CCE powder (3 g) was dissolved in 30 mL of distilled water and heated at 80 °C for 3 h. The obtained solution was filtered and centrifuged at 12,000*g* for 15 min at 4 °C. The obtained supernatant was precipitated overnight at 4 °C by adding ethanol (four times greater than the volume of extract solution), followed by centrifugation at 4500*g* for 10 min. The precipitate was dissolved in 20 mL of distilled water and deproteinized by the Sevag reagent (chloroform/butanol 4:1, v/v), as described by Navarini et al. (1999). The resulting aqueous fraction was extensively dialyzed against double-distilled water for three days and precipitated again by adding fourfold volume of ethanol. After centrifugation, the precipitate was washed with anhydrous ethanol, dissolved in distilled water and lyophilized.

#### Fourier transforms infrared spectroscopic analysis of CCE polysaccharide

The CCE polysaccharides were identified using a Fourier transform infrared spectrophotometer (Shimadzu, FTIR-8400S, Kyoto, Japan) equipped with an IR solution 1.10 Shimadzu software in the range of 4000–500 cm^−1^. FT-IR scans were collected on completely dried thin films of FP cast on KBr discs. The spectra covered the infrared region of 4000–500 cm^−1^, the number of scans per experiment was 10 and the resolution was 6 cm^−1^.

#### Extraction and HPLC analysis

CCE extract (1 g) was mixed with 10 mL of 80% methanol agitated for 10 min, vortexed and then centrifuged at 10,000*g* for 10 min. An aliquot of CCE (0.5 mL) was added to 0.5 mL of acetone and agitated for 30 min at room temperature. After that, the homogenate was centrifuged (12,000*g* for 15 min).

HPLC analysis was carried out with an analytical HPLC system Varian Pro Star model 230 (Varian Associates, Walnut Creek, CA) equipped with a ternary pump (model Q2 Prostar 230) and a photodiode array detector (model Prostar 335). The HPLC separation of the active compounds was carried out on C-18 reverse phase HPLC column (Zorbax, 250 mm ×4.6 mm, particle size 5 μm). The mobile phase consisted of water:acetic acid (98:2 v/v) (A) and water:acetonitrile:acetic acid (58:40:2 v/v/v) (B). The elution gradient used was: 0–80% B for 25 min, 80–100% B for 10 min and 100–0% B for 5 min. The flow rate was 0.9 mL/min and the injection volume was 20 μL. Compound identification was performed at 280 nm for gallic acid, catechin, caffeic acid, epicatechin, vanillic acid and coumarin and at 360 nm for rutin, isorhamnetin, quercetin and kampferol. The identification of all compounds was carried out by comparing their retention times with those obtained by injection of the standard solutions under the same conditions.

### *In vitro* antioxidant activity of CCE

#### DPPH radical-scavenging activity

Antiradical activity was evaluated by measuring the scavenging activity of CCE on the 2,2-diphenyl-l-1-picrylhydrazil (DPPH) radical using the method described by Kirby and Schmidt (1997) with some modifications. Briefly, 500 μL of CCE at different concentrations ranging from 0.05 to 0.6 mg/mL was added to 375 μL of methanol and 125 μL of a DPPH solution (0.2 mM in methanol) as a source of free radicals. These solution mixtures were kept in the dark for 60 min. Scavenging activity was measured by monitoring the decrease in absorbance at 517 nm. BHT (butylated hydroxytoluene) was used as a positive compound. The DPPH radical-scavenging activity (RSA) was calculated using the following formula:

RSA(%)=[(Ac-As)/Ac]×100

where Ac is the absorbance of the control reaction and As is the absorbance of CCE. The IC_50_ values were calculated from the graph plotting. The test was carried out in triplicate.

#### Reducing power assay

The reducing power of the CCE was determined by assessing its ability to reduce iron (III) as described by the method of Yildirim et al. (2001). Briefly, 1.25 mL of phosphate buffer (0.2 M, pH 6.6) was mixed with 1.25 mL of potassium ferricyanide solution (10 g/L) and 1 mL of CCE at different concentrations (0.1–0.7 mg/mL). The mixtures were incubated at 50 °C for 30 min, then 1.25 mL of 10% (w/v) trichloroacetic acid was added and subsequently centrifuged at 3000*g* for 10 min, followed by mixing 1.25 mL of the supernatant solution with 1.25 mL of distilled water and 0.25 mL of ferric chloride (1 g/L). After 10 min, the absorbance was measured at 700 nm. A higher absorbance indicates a higher reducing power.

The EC_50_ value (mg/mL) was the CCE concentration at which the absorbance was 0.5 for the reducing power and was calculated from the graph plotting. BHT was used as a standard, and the test was carried out in triplicate.

#### Metal (Fe^2+^) chelating activity

The chelating ability of Fe^2+^ ions with CCE extract was evaluated using the method of Dinis et al. (1994). A volume of 0.5 mL of CCE extract at different concentrations ranging from 0.1 to 0.8 mg/mL was added to 1.6 mL demineralized water and 0.5 mL of FeCl_2_ (2 mM). After 15 min, 0.1 mL ferrozine (5 mM) was added to the mixture. After 10 min, the absorbance of the complex (Fe^2+^/ferrozine) having a red or purple colour was measured at 562 nm. The chelating activity of mixture Fe^2+^/ferrozine was calculated as:

Chelating power (%)=[(Ac-As)/Ac]×100

Ac is the absorbance of control reaction and As is the absorbance of CCE extract. The EC_50_ value was defined as the concentration (mg/mL) and was calculated from the graph plotting. Ethylenediamine tetraacetic acid (EDTA) was used as a positive control and the test was carried out in triplicate.

#### Animals and treatments

Two-month-old healthy male Wistar rats (*n* = 24) weighing about 120 ± 10 g were purchased from Central Pharmacy of Tunis (Tunisia) and maintained for an adaptation period of 1 weeks under the same conditions of temperature (22 ± 2 °C), relative humidity (70 ± 4%) and 12 h light/dark cycle. The animals were fed commercial pellet diet and tap water *ad libitum*. The animals were treated according to the Tunisian code of practice for the Care and Use of Animals for Scientific Purposes and the European convention for the protection of vertebrate animals used for experimental and other scientific purposes (Council of Europe No123, Strasbourg, 1985). After the adaptation period, animals were divided into four groups of six rats each and treated as follows:

Group 1 (C): control rats given distilled water (0.5 mL/100 g of body weight; i.p.).

Group 2 (Li): rats administered intraperitoneally (i.p.) with 25 mg/kg of lithium carbonate (dissolved in distilled water) twice daily for 30 days. This concentration was chosen according to previous data (Oktem et al. 2005).

Group 3 (CCE): rats given CCE at 100 mg/kg of b.w. for 60 days and then injected with distilled water (0.5 mL/100 g b.w.; i.p.) during the last 30 days of CCE treatment.

Group 4 (Li + CCE): rats given CCE at 100 mg/kg of b.w. for 60 days and then injected with lithium carbonate at a dose 25 mg/kg of b.w. (i.p.) during the last 30 days of CCE treatment.

After 60 days of treatment, animals from each group were rapidly sacrificed by decapitation in order to minimize the handling stress, and their blood samples were collected in two tubes. The first tube was dry and served for serum collection; the second was heparinized and served to determine haematological parameters.

#### Preparation of liver extracts

About 1 g of liver was cut into small pieces and immersed into a 2 mL ice-cold lyses buffer (TBS, pH 7.4) using ultra-turraks homogenizer for 15 min, then filtered and centrifuged (5000*g*, 30 min, 4 °C). Supernatants were stored at −80 °C until use.

### Biochemical assays

#### Evaluation of lipid peroxidation

The lipid peroxidation level in liver was measured as the amount of thiobarbituric acid reactive substance (TBARS) according to the method of Ohkawa et al. (1979). For this assay, 125 μL of supernatant (S1) were mixed with 50 μL of TBS, 125 μL of TCA-BHT to precipitate proteins and centrifuged (1000*g*, 10 min, 4 °C). Then, 200 μL of supernatant was mixed with 40 μL of HCl (0.6 M) and 160 μL of TBA (0.72 mM) dissolved in Tris and the mixture was heated at 80 °C for 10 min. The absorbance was measured at 532 nm. The amount of MDA was calculated using an extinction coefficient of 156 mM ^1 ^cm^−1^ and expressed in nmoles/mg protein.

#### Determination of catalase activity (CAT)

Catalase activity was measured according to the method of Aebi (1984). For the assay, 780 μL of PBS (100 mM, pH 7.4) and 20 μL of (liver) homogenate were taken in a cuvette. The reaction was started by adding 200 μL H_2_O_2_ (500 mM), and absorbance was recorded at every second for 1 min at 240 nm. Enzyme activity was calculated using an extinction coefficient of 0.043 mM^−1 ^cm^−1^ and expressed in international units (I.U.); in micromoles H_2_O_2_ destroyed/min/mg protein at 25 °C.

#### Determination of superoxide-dismutase (SOD)

Liver tissue was homogenized in 10 volumes of ice-cold 1.15% KCl buffer containing 0.4 mM of PMSF and then centrifuged at 2000 rpm for 10 min (4 °C). Total (Cu, Zn and Mn) SOD activity was determined according to the method of Durak et al. (1993). This assay was based on the inhibition of nitro blue tetrazolium (NBT) reduction by the xanthine–xanthine oxidase system as a superoxide generator. One unit of SOD was defined as the enzyme amount causing 50% inhibition in the NBT reduction. SOD activity was expressed as units/mg protein.

#### Determination of gluthatione peroxydase (GPx)

GPx activity was assayed using the method described by Flohe and Gunzler (1984). The enzyme activity was expressed as μmoles of GSH oxidized/min/g protein.

#### Protein assays

Protein content was estimated according to Lowry’s method (1951), using the bovine serum albumin as standard.

#### Determination of haematological parameters

The heparinized blood samples were analyzed in order to determine the haematological parameters (red blood cells, white blood cells, haemoglobin, mean corpuscular volume and haematocrit) using an electronic automatic apparatus (MAXM, Beckman Coulter Inc., Fullerton, CA).

#### Assays of serum markers

The level of glucose, cholesterol, triglycerides as well as the activity of aspartate amino transferase (AST), alanine amino transferase (ALT), lactate dehydrogenase (LDH) and alkaline phosphatase (ALP) in serum were assayed spectrophotometrically using kits (Spinreact, Girona, Spain).

#### Histopathological examination

Liver tissues were quickly excised and immersed for 48 h at 4 °C in a fixative solution (10% formaldehyde, in phosphate buffer, pH 7.6), dehydrated in ethanol and embedded in paraffin. Paraffin sections were cut into ∼5–8 μm using a microtome and stained with haematoxylin-eosin solution (H&E). Tissue preparations were observed and micro-photographed under a light BH_2_ Olympus microscope (Olympus, Tokyo, Japan).

### Statistical analysis

Data were expressed as means ± standard deviation (SD). Statistical significance was assessed by Student’s *t*-test, *p* < 0.05 was considered statistically significant.

## Results

### Phytochemical studies of CCE

#### Total polyphenol and flavonoid contents

The CCE used in this study contained phenolic compounds (125.01 ± 0.90 mg GAE/g CCE) whose level was expressed as gallic acid equivalents. Total flavonoids were expressed as quercetin equivalent per gram of the extract. The flavonoid compounds found in the CCE amounted to 71.02 ± 0.757 mg QE/g CCE (Table 1).

**Table 1.** Total phenolic and total flavonoid contents of cactus cladode extract.

Metabolites	Cactus cladode extract
TPC^a^ (mg GAE/g CCE)	125.01 ± 0.90
TFC^b^ (mg QE/g CCE)	71.02 ± 0.757

Data are expressed as mean ± standard deviation (*n* = 3) are significantly (*p* < 0.05).

a Total phenolic content as gallic acid equivalent.

b Total flavonoid content as quercetin equivalent.

#### HPLC analysis of CCE

**Figure 1.**
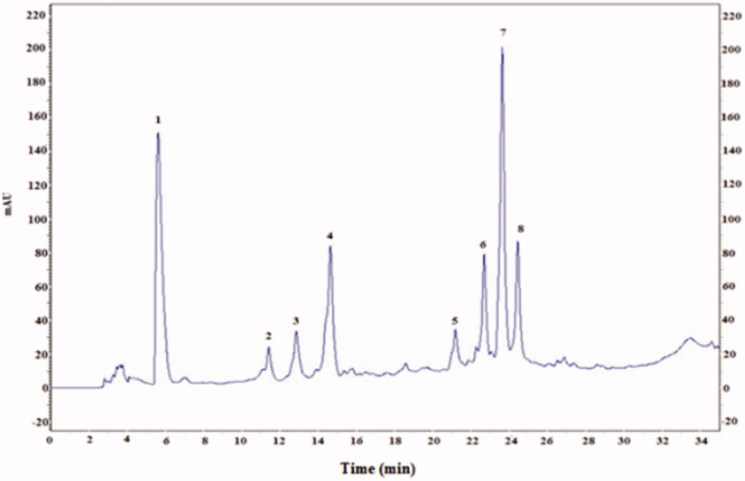
HPLC profile of phenolic acids (*λ =* 280 nm) from cactus (*Opuntia ficus-indica*) cladodes (CCE). Six known phenolic acids identified in CCE: gallic acid (1), catechin (4), caffeic acid (5), epicatechin (6), vanillic acid (7) and coumarin (8). The HPLC separation of the active compounds was carried out on C-18 reverse phase HPLC column (Zorbax, 250 mm ×4.6 mm, particle size 5 μm) on an elution gradient at 25 °C. The mobile phase consisted of water:acetic acid (98:2 v/v) (A) and water:acetonitrile:acetic acid (58:40:2 v/v/v) (B). The elution gradient used was: 0–80% B for 25 min, 80–100% B for 10 min and 100–0% B for 5 min. The flow rate was 0.9 mL/min and the injection volume was 20 μL.

**Figure 2.**
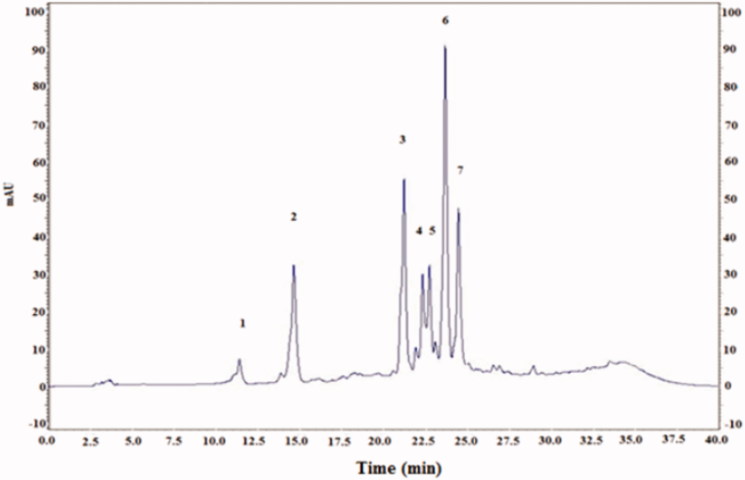
HPLC profile of flavonoids (*λ =* 360 nm) from cactus (*Opuntia ficus-indica*) cladodes (CCE). Four known flavonoids identified in CCE: rutin (2), isorhamnetin (3), quercetin (6), kampferol (7). The HPLC separation of the active compounds was carried out on C-18 reverse phase HPLC column (Zorbax, 250 mm ×4.6 mm, particle size 5 μm) on an elution gradient at 25 °C. The mobile phase consisted of water:acetic acid (98:2 v/v) (A) and water:acetonitrile:acetic acid (58:40:2 v/v/v) (B). The elution gradient used was: 0–80% B for 25 min, 80–100% B for 10 min and 100–0% B for 5 min. The flow rate was 0.9 mL/min and the injection volume was 20 μL.

The HPLC elution profile of CCE shown in Figure 1 revealed the presence of phenolic acids identified at 280 nm. There were six known phenolic acids found in CCE (gallic acid, catechin, caffeic acid, epicatechin, vanillic acid, and coumarin) and two unknown compounds. The HPLC analysis of flavonoids showed seven compounds identified at 360 nm including four known flavonoids: rutin, isorhamnetin, quercetin and kampferol (Figure 2).

#### FT-IR spectral analysis of CCE polysaccharides

**Figure 3.**
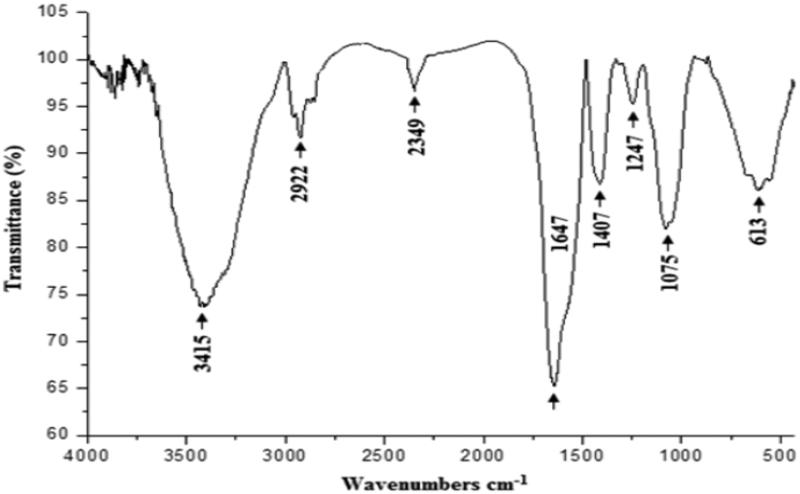
Infrared spectra of the polysaccharide extracted from CCE recorded in the frequency range of 4000–500 cm^−1^.

As shown in Figure 3, the FT-IR spectra of CCE-purified polysaccharide displayed a broad stretching intense peak at 3415 cm^−1^, which is the characteristic absorption of hydroxyl groups, followed by weak C–H stretching bands at 2922 cm^−1^ (Xu et al. 2009). The weak peak at 2349 cm^−1^ is a non-identified compound. The band around 1647 cm^−1^ was attributed to the stretching vibration of C = O in protonated carboxylic acid. It also revealed the presence of uronic acids. The band towards 1407 cm^−1^ was attributed to the absorbance of the COO^−^ deprotonated carboxylic group (Manrique & Lajolo 2002). The peak observed at 1247 cm^−1^ could be explained by the C–O stretching band of complex polysaccharides (Naqvi et al. 2011). The peak observed at 1075 cm^−1^ could be characteristic of rhamnose polysaccharide content and the peak observed at 613 cm^−1^ could be characteristic of β-d-glucose (Zhao et al. 2007).

### Antioxidant capacities of CCE

#### DPPH radical-scavenging activity

**Figure 4.**
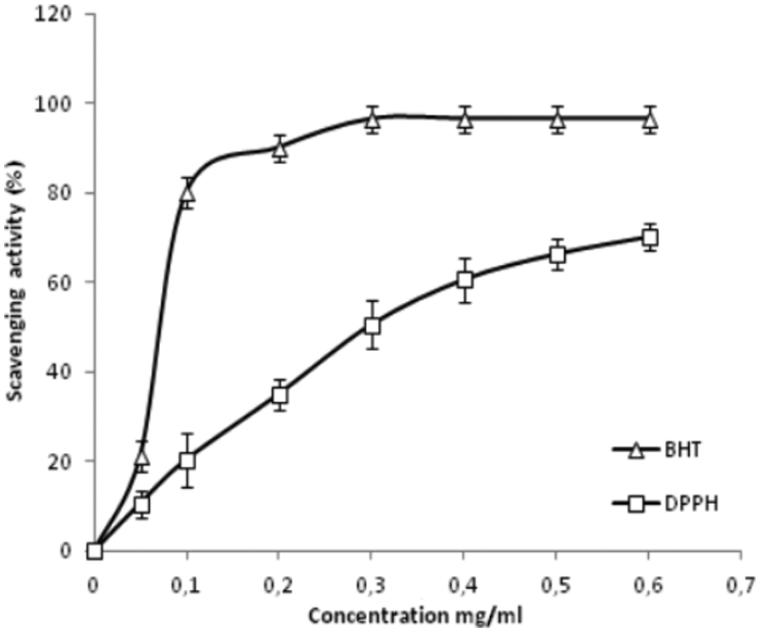
DPPH-radical-scavenging activity of CCE and positive control BHT at different concentrations. Data are expressed as mean ± standard deviation of the mean (*n =* 3).

As can be seen in Figure 4, CCE free radical-scavenging activities were able to reduce the stable free radical DPPH presented by IC_50_ value and defined as the concentration of the antioxidant required to scavenge 50% of DPPH present in the test solution. The value of CCE extract was 0.30 ± 0.03 mg/mL. The results were compared with the scavenging ability of control samples of BHT (0.09 ± 0.06 mg/mL).

#### Reducing power assay

Based on the reducing power assay, it was found that the addition of CCE led to the reduction of Fe^3+ ^to Fe^2+ ^by donating an electron The effective concentration of CCE EC_50_ (0.36 ± 0.08 mg/mL) yielded 0.5 of absorbance compared with BHT (0.039 ± 0.05 mg/mL) as a positive control (Table 2).

**Table 2.** Reducing power and Fe^2+ ^chelating activity of cactus cladode extract.

Compounds	IC_50_ reducing power (mg/mL)	IC_50_ (Fe^2+^) chelating activity (mg/mL)
Cactus cladode extract	0.36 ± 0.08	0.49 ± 0.03
BHT	0.039 ± 0.05	–
EDTA	–	0.034 ± 0.01

Values are expressed as mean ± standard deviation (*n* = 3) are significantly (*p <* 0.05).

#### Metal (Fe^2+^) chelating activity

The metal chelating activity was evaluated by measuring the formation of the complex ferrozine – Fe^2+^. Results presented in Table 2 show high-level chelating activity of CCE (IC_50_= 0.49 mg/mL), but was not stronger than the standard EDTA (IC_50 _=_ _0.034 mg/mL).

#### Evaluation of lipid peroxidation (MDA) and antioxidant enzymes (SOD, GPx and CAT)

**Figure 5.**
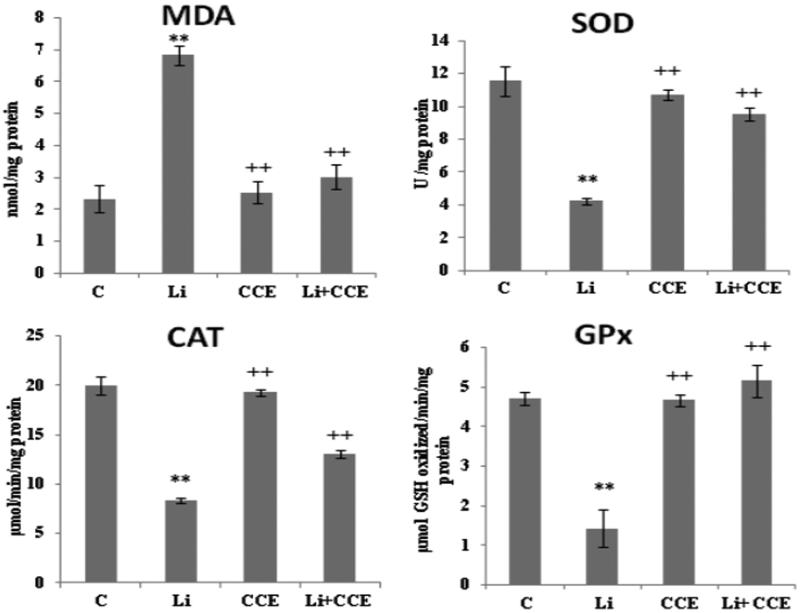
Effect of lithium carbonate and CCE on MDA level and antioxidant enzyme activities (SOD, CAT and GPx) in control (C), carbonate lithium-treated (Li), cactus-treated (CCE), and cactus supplemented with carbonate lithium (Li + CCE) groups. Data are expressed as means ± SD for six rats in each group. Statistical comparison was performed using Student’s *t*-test. *p < 0.05, **p < 0.01 compared with control group (C). +*p* < 0.05, ++*p* < 0.01 compared with lithium carbonate (Li)-treated group.

The results presented in Figure 5 shows the effect of lithium alone and in combination with CCE on the induction of lipid peroxidation in liver determined on the basis of MDA level. The latter significantly increased in liver (*p* < 0.01) in lithium-treated rats compared to controls (3.02 ± 0.36 nmol nmol/mg protein), which suggested the presence of oxidative stress. Administration of CCE (*p* < 0.01) led to a significant reduction in MDA levels compared to the lithium-treated group (6.85 ± 0.31/mg protein).

Figure 5 also shows that treatment with Li induced a significant (*p* < 0.01) decrease in SOD, CAT and GPx. These changes were alleviated when the rats were treated with CCE; there was significant increase in the activity of these enzymes to almost control values. On the other hand, in CCE-treated rats, no significant change in enzymes activities occurred when compared with that of normal group.

#### Haematological parameters

The effects of lithium, CCE and their combination on haematological parameters in the rats are shown in Table 3. Results indicated that Li caused a significant reduction *(p* < 0.01) of RBC, Hb, Ht, VCM values and decreased WBC counts compared to the control group. In the case of the group treated with both lithium and CCE at 100 mg/kg of b.w., the haematological parameters were found to revert to almost normal values.

**Table 3.** Change in hematological parameters of control and rats treated with lithium carbonate (Li), CCE or their combination (CCE + Li).

	Treatment groups			
Parameters	C	Li	CCE	CCE + Li
RBC (10^6^/μL)	7.99 ± 0.4	6.08 ± 0.1^b^	8.10 ± 0.2^d^	7.18 ± 0.2^d^
WBC (10^3/^μL)	10.05 ± 0.5	18.75 ± 0.1^b^	10.50 ± 0.6^d^	12.10 ± 0.2^d^
Hb (g/dl)	14.80 ± 0.1	12.25 ± 0.5^b^	15.12 ± 0.66^d^	14.07 ± 0.1^d^
VCM (10^−6^μm^3/^RBC)	46.50 ± 0.9	23.40 ± 0.6^b^	45.02 ± 0.9**+**^d^	39.00 ± 1.1^d^
Ht (%)	43.07 ± 1.3	37.05 ± 0.58^b^	44.25 ± 0.61^d^	41.45 ± 0.6^d^

RBC: red blood cell; WBC: white blood cell; Hb: Hemoglobin; VCM: mean corpuscular volume; Ht: Haematocrit. Data are expressed as means ± standard deviation (SD) for six rats in each group. Statistical comparison was performed using Student’s test.

b *p* < 0.01 compared with control group (C).

d *p* < 0.01 compared with lithium carbonate-treated group (Li).

#### Serum markers of liver damage

Lithium treatment induced severe liver damage evidenced in serum by a significant increase (*p* < 0.01) in the levels of glucose (7.02 ± 0.41 mmol/L), cholesterol (2.87 ± 0.12 mmol/L), triglycerides (1.74 ± 0.05 mmol/L), AST (345 ± 0.3 U/L), ALT (122 ± 0.2 U/L), LDH (1101 ± 5.2 U/L) and ALP (85 ± 2.9 U/L). However, these biochemical parameters were significantly reduced (*p* < 0.01) in rats treated with a combination of CCE and Lithium compared to the lithium-treated group (5.46 ± 0.46 mmol/L, 1.20 ± 0.10 mmol/L and 1.46 ± 0.10 mmol/L). Also, administration of CCE alone does not affect the parameters (Table 4).

**Table 4.** Assay of serum markers of control and rats treated with lithium carbonate (Li), CCE, or their combination (CCE + Li).

	Treatment groups			
Parameters	C	Li	CCE	CCE + Li
Glucose (mmol/L)	4.85 ± 0.25	7.02 ± 0.41^b^	4.99 ± 0.41^d^	5.46 ± 0.46^d^
Cholesterol (mmol/L)	1.40 ± 0.06	2.87 ± 0.12^b^	1.43 ± 0.09^d^	1.46 ± 0.25^d^
Triglycerides (mmol/L)	1.08 ± 0.1	1.74 ± 0.05^b^	1.07 ± 0.1^d^	1.16 ± 0.3^d^
AST (U/L)	150 ± 0.9	345 ± 0.3^b^	166 ± 0.1^d^	240 ± 1.4^d^
ALT (U/L)	71 ± 0.08	122 ± 0.2^b^	68.5 ± 1.1^d^	85.4 ± 0.9^d^
LDH (U/L)	877 ± 8.2	1101 ± 5.2^b^	872 ± 2.4^d^	845 ± 4.1^d^
ALP (U/L)	54 ± 8.1	85 ± 2.9^b^	56 ± 1.73^d^	50 ± 4.50^d^

AST: Aspartate aminotransferase; ALT: Alanine aminotransferase; LDH: lacatate dehydrogenase; ALP: alkaline phosphatase. Data are expressed as mean ± standard deviation (SD) for six rats in each group. Statistical comparison was performed using Student’s test.

b *p* < 0.01 compared with control group (C).

d *p* < 0.01 compared with lithium carbonate-treated group (Li).

### Histological observations

**Figure 6.**
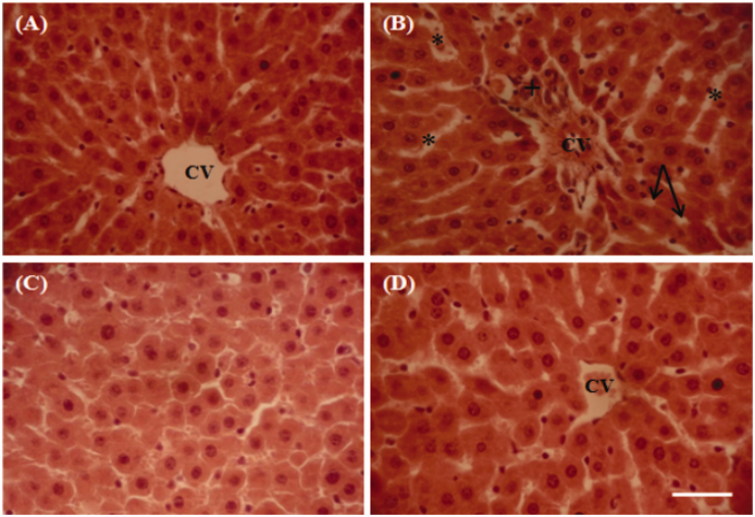
Representative photographs from the liver showing the protective effect of cactus cladode extract on lithium carbonate induced hepatic injury in rats. (A) Controls, (B) rats treated with lithium carbonate, (C) rats treated with cactus cladode extract and (D) rats treated with the combination of cactus cladode extract and lithium carbonate. Liver sections were stained using the haematoxylin-eosin method. Original magnifications: ×400; CV: central vein in the liver + congested central veins; *sinusoidal dilatation; vacuolization: inflammatory cell infiltration.

The liver showed the following histopathological changes (Figure 6). The control group shows no obvious abnormality (Figure 6(A)). In the present study, lithium application constituted histopathological changes which caused severe liver damage, including sinusoidal dilation, congested central veins, vacuolization and inflammatory cell infiltration when compared with control liver (Figure 6(B)). The hepatocellular damage was slightly reduced in lithium and CCE-treated rats at a dose of 100 mg/kg b.w. (Figure 6(D)). However, no histological alterations were observed in the liver of CCE-treated group when compared to the control (Figure 6(C)).

## Discussion

Over the past several years, *Opuntia ficus-indica* has been widely used in traditional herbal medicine to treat various types of potential damage to vital organs due to the protective activities and nutritional values this plant possesses. In the present work, the CCE was phytochemically studied in order to evaluate the total phenolic and flavonoid content in CCE and investigate its antioxidant potency against oxidative stress induced in rats by the injection of lithium carbonate (Li_2_CO_3_). It is well-known that phenolic substances found in CCE exhibit considerable free radical-scavenging activities by virtue of their reactivity as hydrogen- or electron-donating agents, as well as metal ion-chelating properties, preventing metal-induced free radical formation (Rice-Evans et al. 1996). As shown in Table 1, the plant contained high phenolic and flavonoid content. These results are more important than those reported in previous studies showing the antioxidant and antigenotoxic activities of CCE (Brahmi et al. 2011). Also, it was found that several analytical methods such as free radical scavenging, reducing capacity and metal chelating activity that have been used to evaluate the eventual antioxidant capacity of CCE *in vitro* could be employed altogether to evaluate hepatoprotective effects *in vivo*. The stable DPPH is widely used to investigate the scavenging activity of some natural compounds due to their hydrogen donating ability (Hajji et al. 2010). As shown in Figure 4, the CCE was able to effectively reduce the stable free radical DPPH. These results suggested that the presence of phenolic compounds in CCE might be the main cause of their considerable radical-scavenging activity. However, the phenolic compounds are highly dependent on the number and arrangements of hydroxyl groups as well as the presence of constituents to serve as electron donors (Lapornik et al. 2005). Table 2 shows that the reducing power assay used to evaluate CCE antioxidant potential is based on the reduction of Fe^3+ ^to Fe^2+ ^to donate electrons (Yildirim et al. 2001). This assay indicated that the CCE contained a high amount of total phenolics and flavonoids that showed greater reducing power than that of synthetic antioxidant (BHT). Metal chelating activity is claimed to be among antioxidant mechanisms since it reduces the concentration of the catalyzing transition metal in lipid peroxidation. Among the transition metals, Fe^2+^ ion is known to be the most important lipid prooxidant due to its high reactivity. IC_50_ value of chelating activity was the concentration of the CCE required to chelate 50% of Fe^2+^ present in the reaction mixtures. Lower IC_50_ reflected better chelating activity. Results here showed that CCE had the highest chelating activity compared with EDTA as a positive standard.

For the first time, these antioxidant properties could render CCE an excellent plant to protect lithium-induced toxicity *in vivo*. Therefore, this alkali metal is known to enhance the production of reactive oxygen species (ROS) leading to an oxidative stress in different organs and can inflict damage on lipids, proteins and DNA (Khairova et al. 2012; Vijaimohan et al. 2010). Previous studies have revealed that exposure of rats to lithium toxicity can lead to alteration of antioxidant defence mechanisms and enhancement of lipid peroxidation in rats (Nciri et al. 2012; Oktem et al. 2005). Under our experimental conditions, daily injection of Li at a dose of 25 mg/kg twice for 30 days caused a significant increase in MDA levels (*p* < 0.01) in liver tissues, which acted as a potential lipid peroxidation (LPO) biomarker. Increase in MDA level enhanced the LPO and increased ROS production with subsequent disturbance of membrane function and integrity (Toplan et al. 2013). Administration of CCE at a dose of 100 mg/kg of b.w. prevented this lithium-induced increase of MDA level when compared with the lithium-treated rats. This action could be explained by the ability of CCE to reduce the LPO level in cell membrane by scavenging free radicals induced by lithium. In this context, other works have demonstrated that CCE is capable of protecting tissues against oxidative stress induced by various toxins by inhibiting LPO both *in vivo* and *in vitro* (Brahmi et al. 2012; Hfaiedh et al. 2014). The mechanisms whereby lithium exerts its deleterious effects have not been accurately determined yet. However, it has been suggested that induction of oxidative stress is the central mechanism whereby lithium exert their cellular effect (Nciri et al. 2012; Oktem et al. 2005). Additionally, it has been previously reported that acute exposure of rats to Li may induce oxidative stress by excessive formation of reactive oxygen species (Khairova et al. 2012; Musik et al. 2014).

Reduction in antioxidant enzymes (SOD, CAT and GPx) is responsible for lithium-induced oxidative damage (Khairova et al. 2012; Vijaimohan et al. 2010). In available literature data, Li has been found to enhance GPx activities. Our results further confirmed those in earlier studies, indicating that oral administration of Li_2_CO_3_ in drinking water for 4 weeks leads to a decrease in GPx activity in different organs (Kielczykowska et al. 2008; Vijaimohan et al. 2010). In addition, a significant reduction in CAT activity was noticed in Li-treated rats, which could be explained by the overconsumption of this enzyme involved in the conversion of H_2_O_2_ to H_2_O. As for the antioxidant enzyme SOD, it showed a significant decrease in liver after lithium treatment, which resulted in the loss of free radical scavenging mechanism (Keen et al. 1985). SOD is also known for its role in inhibiting OH^−^ production by scavenging O_2_^−^, and thus leads to a decrease in the initiation of LPO (Fridovich 1994).

The administration of CCE had a potent protective effect on oxidative lithium-induced damage in rats, as revealed by a significant increase in hepatic CAT, SOD and GPx activities. Also, the beneficial effect of CCE could be explained by the antioxidant capacity of its constituents. As can be seen in Figures 1 and 2, the HPLC analysis revealed the presence of four flavonoids (rutin, isorhamnetin, quercetin, kampferol) and six phenolic acids (gallic acid, catechin caffeic acid, epicatechin, vanillic acid, coumarin), which are known to have beneficial effects as their responsibility in preventing the formation of reactive oxygen species (Alimi et al. 2013). Further, the antioxidant capacity of CCE was found to be due to its total polyphenolic contents as well as polysaccharides (fibres). A study by Lee et al. (2002) indicates that the antioxidant properties of CCE are mainly due to vitamins and flavonoids, more particularly quercetin that has been reported to be a highly efficient radical scavenger.

Lithium has also been shown to induce haemorrhage, inflammatory cells infiltration congestion and sinusoidal dilatation, which can cause increased WBC counts. The elevated WBC counts could be due to the stimulation of immune system and showed that there were oedema and inflammation (Kielczykowska et al. 2014; Sharma & Iqbal 2005). This study also indicated that exposure to Li induced significant haematological changes because of decrease in RBC, Hb, Ht and VCM compared to control, which suggested the occurrence of anaemia (Kielczykowska et al. 2014; Malhotra & Dhawan 2008). The significant reduction in RBC counts, Hb and Ht might be due to diminished erythropoietin, haemoglobin synthesis reduction and an increase in erythrocyte destruction rate in hematopoietic tissues. Therefore, the observed decrease of these parameters giving rise to the production of oxidative radicals could reach the cell membrane and cause membrane lipid peroxidation (Kato et al. 2006).

The obtained result is in agreement with Malhotra and Dhawan (2008), who point out that alkali metal exposure promotes oxidative damage in erythrocytes. However, supplementation with CCE exhibited potential protective effect in most of the haematological parameters in lithium-treated rats (Table 4). Therefore, the CCE was able to reactivate the erythropoiesis mechanism and thus enhance the production of erythropoietin. CCE was found to have important antioxidant properties able to inhibit Li-induced haematological changes. Also, we believe that the CCE examined in earlier studies has anti-inflammatory effects (Antunes-Ricado et al. 2015).

Additionally, the experimental results also revealed that the lithium treatment could affect the lipid metabolism of liver (total cholesterol and triglycerids) and glucose level in rats. The increase of these parameters can be seen as a sign of liver damage. Similar changes have been reported by Vijaimohan et al. (2010) who point out that lithium induced free radicals, which is likely to cause change in serum lipid profiles. Glucose is considered as a parameter more vulnerable to the presence of lithium in an organism influencing carbohydrate metabolism (Kielczykowska et al. 2014). An increased level of glucose was also noticed in rats after oral administration of Li_2_CO_3_ with different doses for 7 weeks (Sharma & Iqbal 2005). Also, intravenous lithium causes an increase in plasma glucose and reduction of plasma insulin in normal rats (Kielczykowska et al. 2014). Moreover, lithium seemed to produce other biochemical defects in liver revealed by a significant rise in serum hepatic marker enzymes, such as AST, ALT, LDH and ALP (Musik et al. 2014; Vijaimohan et al. 2010). Both enzymes AST and ALT can be used in order to elevate the function and integrity of liver cells inducing an increase in the serum levels (Ahmad et al. 2011; Kielczykowska et al. 2014). Additionally, the increase of LDH levels is considered an indicator of liver damage (Ncibi et al. 2008). Apart from LDH, the elevated level of ALP reflected a cytotoxic effect exerted by lithium. In this context, several authors have reported the significant elevation in these serum enzyme levels after lithium treatment (Nordenberg et al. 1982). Pretreatment with CCE significantly restored the increase in serum hepatic enzymes in lithium-treated rats when compared with lithium-intoxicated rats. Earlier studies in this laboratory, which have shown a remarkable enhancement in the different parameters studied in rats treated with nickel (Hfaiedh et al. 2008) and chlorpyrifos (Ncibi et al. 2008), are in agreement with the results obtained in the present study. This action may be due to the ability of CCE and its bioactive constituents to stabilize the membrane permeability and reduce the leakage of enzymes into the blood (Alimi et al. 2013). In order to further confirm the protective effect of CCE against lithium-induced oxidative damage in liver, a histopathological examination was performed (Figure 6). In fact, histological changes, seen in the liver of animals treated with lithium, are characterized by an important sinusoidal dilation, congested central veins, vacuolization and inflammatory cell infiltration. Our results corroborated previous findings reported by Sharif et al. (2011), who indicated that alkali metal causes histopathological and enzymatic changes in rats. However, the CCE attenuated the histological alterations induced in lithium-treated rats, which could be associated with CCE antiradical/antioxidant and metal-chelating capacities (Dok Go et al. 2003).

## Conclusion

In the present work, the CCE under investigation was found to possess excellent antioxidant activities based on various *in vitro* and *in vivo* assays. The different *in vitro* antioxidant tests proved that the CCE is rich in flavonoids, phenolic compounds as well as polysaccharide. Also, this study demonstrated that CCE could have a protective effect on lithium-induced hepatotoxicity and oxidative stress in our experimental model. Future research is needed to carry out further biochemical investigations in order to isolate and clarify the mechanism behind the activity of this extract.

## References

[CIT0001] Aebi A. 1984 Catalase *in vitro* . Meth Enzymol. 105:121–126. 6727660 10.1016/s0076-6879(84)05016-3

[CIT0002] Ahmad M, Elnakady Y, Farooq M, Wadaan M. 2011 Lithium induced toxicity in rats: blood serum chemistry, antioxidative enzymes in red blood cells and histopathological studies. Biol Pharm Bull. 34:272–277. 21415540 10.1248/bpb.34.272

[CIT0003] Alimi H, Hfaeidh N, Bouoni Z, Mbarki S, Sakly M, Ben Rhouma K. 2013 Cactus (*Opuntia ficus indica f. inermis*) fruit juice protects against ethanol-induced hematological and biochemical damages in rats. Afr J Biotechnol. 12:7099–7105.

[CIT0004] Alimi H, Hfaeidh N, Bouoni Z, Sakly M, Rhouma KB. 2013 Ameliorative effect of *Opuntia ficus indica* juice on ethanol-induced oxidative stress in rat erythrocytes. Exp Toxicol Pathol. 65:391–396. 22285760 10.1016/j.etp.2011.12.003

[CIT0005] Alimi H, Hfaiedh N, Bouoni Z, Sakly M, Ben Rhouma K. 2011 Evaluation of antioxidant and antiulcerogenic activities of *Opuntia ficus indica f. inermis* flowers extract in rats . Environ Toxicol Pharmacol. 32:406–416. 22004960 10.1016/j.etap.2011.08.007

[CIT0007] Antunes-Ricado M, Gutiérrez-Uribe JA, Martínez-Vitela C, Serna-Saldívar SO. 2015 Tropical anti-inflammatory effects of isorhamnetin glycosides isolated from *Opuntia ficus-indica* . Biomed Res Int. [Online]. Available from: http://dx.doi:10.1155/2015/847320. 10.1155/2015/847320 PMC4363586 25821823

[CIT0008] Brahmi D, Ayed Y, Hfaiedh M, Bouaziz C, Ben Mansour H, Zourgui L, Bacha H. 2012 Protective effect of cactus cladode extract against cisplatin induced oxidative stress, genotoxicity and apoptosis in balb/c mice: combination with phytochemical composition. BMC Complement Altern Med. 31:12–111. 10.1186/1472-6882-12-111 PMC3567432 22849573

[CIT0009] Brahmi D, Bouaziz C, Ayed Y, Ben Mansour H, Zourgui L, Bacha H. 2011 Chemopreventive effect of cactus *Opuntia ficus indica* on oxidative stress and genotoxicity of aflatoxin B1. Nutr Metab (Lond). 8:73–73. 22008149 10.1186/1743-7075-8-73 PMC3214131

[CIT0010] Dewanto V, Wu X, Adom KK, Adom KK, Liu RH. 2002 Thermal processing enhances the nutritional value of tomatoes by increasing total antioxidant activity. J Agric Food Chem 50:3010–3014. 11982434 10.1021/jf0115589

[CIT0011] Dinis TC, Maderia VM, Almeida LM. 1994 Action of phenolic derivatives (acetaminophen, salicylate, and 5-aminosalicylate) as inhibitors of membrane lipid peroxidation and as peroxyl radical scavengers. Arch Biochem Biophys. 315:161–169. 7979394 10.1006/abbi.1994.1485

[CIT0012] Durak I, Yurtarslanl Z, Canbolat O. 1993 A methodological approach to superoxide dismutase (SOD) activity assay based on inhibition of nitroblue tetazolium (NBT) reduction. Clin Chim Acta. 214:103–104. 8453769 10.1016/0009-8981(93)90307-p

[CIT0013] Flohe L, Gunzler WA. 1984 Assays of glutathione peroxidase. Methods Enzymol. 105:114–121. 6727659 10.1016/s0076-6879(84)05015-1

[CIT0016] Fridovich I. 1994 Superoxide radical and superoxide dismutases . Annu. Rev. Biochem. 64:97–112. 10.1146/annurev.bi.64.070195.000525 7574505

[CIT0016a] Hajji M, Masmoudi O, Souissi N, Triki Y, Kammoun S, Nasri M. 2010 Chemical composition, angiotensin I-converting enzyme (ACE) inhibitory, antioxidant and antimicrobial activities of the essential oil from *Periploca laevigata* root barks. Food Chem. 121:724–731.

[CIT0017] Hfaiedh M, Brahmi D, Zourgui L. 2014 Hepatoprotective effect of *Taraxacum officinale* leaf extract on sodium dichromate-induced liver injury in rats. Environ Toxicol. 31:339–349. 25270677 10.1002/tox.22048

[CIT0018] Hfaiedh N, Allagui MS, Hfaiedh M, Feki AE, Zourgui L, Croute F. 2008 Protective effect of cactus (*Opuntia ficus indica*) cladode extract upon nickel-induced toxicity in rats. Food Chem Toxicol. 46:3759–3763. 18950672 10.1016/j.fct.2008.09.059

[CIT0019] Kato GJ, McGowan V, Machado RF, Little JA, Taylor J, Morris CR, James S, Wang NX, Poljakovic M, Morris SM, et al 2006 Lactate dehydrogenase as a biomarker of hemolysis-associated nitric oxide resistance, priapism, leg ulceration, pulmonary hypertension, and death in patients with sickle cell disease. Blood. 107:2279–2285. 16291595 10.1182/blood-2005-06-2373 PMC1895723

[CIT0020] Keen CL, Tamura T, Lonnerdal B, Hurley LS, Helstud CH. 1985 Changes in hepatic superoxide dismutase activity in alcoholic monkeys. Am J Clin Nutr. 41:929–932. 4039529 10.1093/ajcn/41.5.929

[CIT0021] Khairova R, Pawar R, Salvadore G, Juruena MF, Sousa RT, Soeiro-de-Souza MG, Salvador M, Zarate CA, Gattaz WF, Machado-Vieira R. 2012 Effects of lithium on oxidative stress parameters in healthy subjects. Mol Med Rep. 5:680–682. 22200861 10.3892/mmr.2011.732 PMC3289682

[CIT0022] Kielczykowska M, Kocot J, Kurzepa J, Lewandowska A, Zelazowska R, Musik I. 2014 Could selenium administration alleviate the disturbances of blood parameters caused by lithium administration in rats? Biol Trace Elem Res. 158:359–364. 24676629 10.1007/s12011-014-9952-4 PMC4012153

[CIT0023] Kielczykowska M, Musik I, Pasternak K. 2008 Relationships between silicon content and glutathione peroxidase activity in tissues of rats receiving lithium in drinking water. Biometals. 21:53–59. 17447120 10.1007/s10534-007-9092-9

[CIT0024] King H, Aubert RE, Herman WH. 1988 Global burden of diabetes, 1995–2025: prevalence, numerical estimates, and projections. Diabetes Care. 21:1414–1431. 10.2337/diacare.21.9.1414 9727886

[CIT0025] Kirby AJ, Schmidt RJ. 1997 The antioxidant activity of Chinese herbs for eczema and of placebo herbs-I. J Ethnopharmacol. 56:103–110. 9174970 10.1016/s0378-8741(97)01510-9

[CIT0026] Lapornik B, Prosek M, Wondra AG. 2005 Comparison of extracts prepared from plant by-products using different solvents and extraction time. J Food Eng. 71:214–222.

[CIT0027] Lee JC, Kim HR, Kim J, Jang YS. 2002 Antioxidant property of an ethanol extract of the stem of *Opuntia ficus-indica* var *saboten* . J Agric Food Chem. 50:6490–6496. 12381138 10.1021/jf020388c

[CIT0029] Malhotra A, Dhawan DK. 2008 Zinc improves antioxidative enzymes in red blood cells and hematology in lithium-treated rats. Nutr Res. 28:43–50. 19083387 10.1016/j.nutres.2007.11.002

[CIT0030] Manrique GD, Lajolo FM. 2002 FT-IR spectroscopy as a tool for measuring degree of methyl esterification in pectins isolated from ripening papaya fruit. Post Biol Technol. 25:99–107.

[CIT0031] Musik I, Kocot J, Kiełczykowska M. 2014 Effect of sodium selenite on chosen anti- and pro-oxidative parameters in rats treated with lithium: a pilot study. Pharmacol Rep. 67:446–450. 25933952 10.1016/j.pharep.2014.11.010

[CIT0032] Naqvi SA, Khan MM, Shahid M, Jaskani MJ, Khan IA, Zuber M, Zia KM. 2011 Biochemical profiling of mucilage extracted from seeds of different citrus rootstocks. Carbohydr Polym. 83:623–628.

[CIT0033] Narayana DB, Dobriyalm RM. (2000). Complimentary medicines and 21st century therapeutics: challenges for pharmacologist In: Gupta SK, editor. Pharmacology and therapeutics in the new millennium. New Delhi, India: Narosa Publishing House, pp. 326–335.

[CIT0034] Navarini L, Gilli R, Gombac V, Abatangelo A, Bosco M, Toffanin R. 1999 Polysaccharides from hot water extracts of roasted *Coffea arabica* beans: Isolation and characterization. Carbohydr Polym. 40:71–81.

[CIT0035] Ncibi S, Ben Othman M, Akacha A, Krifi MN, Zourgui L. 2008 *Opuntia ficus indica* extract protects against chlorpyrifos-induced damage on mice liver. Food Chem Toxicol. 46:797–802. 17980473 10.1016/j.fct.2007.08.047

[CIT0036] Nciri R, Allagui MS, Bourogaa E, Saoudi M, Murat JC, Croute F, Elfeki A. 2012 Lipid peroxidation, antioxidant activities and stress protein (HSP72/73, GRP94) expression in kidney and liver of rats under lithium treatment. J Phys Biochem. 68:11–18. 10.1007/s13105-011-0113-3 21948186

[CIT0038] Nordenberg J, Kaplansky M, Beery E, Klein S, Beitner R. 1982 Effects of lithium on the activities of phosphofructokinase and phosphoglucomutase and on glucose-1,6-diphosphate levels in rat muscles, brain and liver. Biochem Pharmacol. 31:1025–1031. 6211175 10.1016/0006-2952(82)90338-0

[CIT0039] Ohkawa H, Ohishi N, Yagi K. 1979 Assay for lipid peroxides in animal tissues by thiobarbituric acid reaction. Anal Biochem. 95:351–358. 36810 10.1016/0003-2697(79)90738-3

[CIT0040] Oktem F, Ozguner F, Sulak O, Olgar S, Akturk O, Yilmaz HR, Altuntas I. 2005 Lithium-induced renal toxicity in rats: protection by a novel antioxidant caffeic acid phenethyl ester. Mol Biol Cell. 277:109–115. 10.1007/s11010-005-5426-5 16132721

[CIT0042] Rice-Evans CA, Miller NJ, Paganga G. 1996 Structure-antioxidant activity relationships of flavonoids and phenolic acids. Free Radic Biol Med. 20:933–956. 8743980 10.1016/0891-5849(95)02227-9

[CIT0043] Rogers MP, Whybrow PC. 1971 Clinical hypothyroidism occurring during lithium treatment: two case histories and a review of thyroid function in patients. Am J Psychiat. 128:158–163. 4107030 10.1176/ajp.128.2.158

[CIT0044] Sahu AK, Gautam MK, Deshmukh PT, Kushwah L, Silawat N, Akbar Z, Muthu MS. 2013 Effect of embelin on lithium-induced nephrogenic diabetes insipidus in albino rats. Asian Pac J Trop Dis. 2:729–733.

[CIT0045] Sharif N, Rabia A, Iftikhar O. 2011 Adverse effects of withdrawal of chronic lithium therapy on liver – a histological study. Pakistan J Zool. 43:1155–1160.

[CIT0046] Sharma SD, Iqbal M. 2005 Lithium induced toxicity in rats: a hematological, biochemical and histopathological study. Biol Pharm Bull. 28:834–837. 15863888 10.1248/bpb.28.834

[CIT0048] Tesoriere L, Fazzari M, Allegra M, Livrea MA. 2005 Biothiols, taurine, and lipid-soluble antioxidants in the edible pulp of Sicilian cactus pear (*Opuntia ficus-indica*) fruits and changes of bioactive juice components upon industrial processing. J Agric Food Chem. 53:7851–7855. 16190641 10.1021/jf050636f

[CIT0049] Toplan S, Dariyerli N, Ozdemir S, Ozcelik D, Zengin EU, Akyolcu MC. 2013 Lithium-induced hypothyroidism: oxidative stress and osmotic fragility status in rats. Biol Trace Elem Res. 152:373–378. 23408263 10.1007/s12011-013-9629-4

[CIT0050] Vijaimohan K, Mallika J, Shyamala DC. 2010 Chemoprotective effect of sobatum against lithium-induced oxidative damage in rats. Pharmacology. 2:68–73. 10.4103/0975-1483.62217 PMC3035889 21331195

[CIT0051] Wiese J, McPherson S, Odden MC, Shlipak MG. 2004 Effect of *Opuntia ficus indica* on symptoms of the alcohol hangover. Arch Intern Med. 164:1334–1340. 15226168 10.1001/archinte.164.12.1334

[CIT0052] Xu W, Zhang F, Luo Y, Ma L, Kou X, Huang K. 2009 Antioxidant activity of a water-soluble polysaccharide purified from *Pteridium aquilinum* . Carbohydr Res. 344:217–222. 19036355 10.1016/j.carres.2008.10.021

[CIT0053] Yildirim A, Mavi A, Kara AA. 2001 Determination of antioxidant and antimicrobial activities of *Rumex crispus* L. extracts. J Agric Food Chem. 49:4083–4089. 11513714 10.1021/jf0103572

[CIT0054] Zhao M, Yang N, Yang B, Jiang Y, Zhang G. 2007 Structural characterization of water-soluble polysaccharides from *Opuntia monacantha* cladodes in relation to their anti-glycated activities. Food Chem. 105:1480–1486.

